# Definition and Predictors of Early Recurrence in Neoadjuvantly Treated Esophageal and Gastroesophageal Adenocarcinoma: a Dual-Center Retrospective Cohort Study

**DOI:** 10.1245/s10434-024-16403-5

**Published:** 2024-11-05

**Authors:** Ingmar F. Rompen, Adrian T. Billeter, Nerma Crnovrsanin, Leila Sisic, Kerstin J. Neuschütz, Julian Musa, Martin Bolli, Lana Fourie, Marko Kraljevic, Mohammed Al-Saeedi, Henrik Nienhüser, Beat P. Müller-Stich

**Affiliations:** 1 Department of General, Visceral and Transplantation Surgery, Heidelberg University Hospital, Heidelberg, Germany; 2 Department of Surgery, Clarunis-University Digestive Health Care Center, St. Clara Hospital and University Hospital Basel, Basel, Switzerland

**Keywords:** Esophageal cancer, Esophagectomy, Early recurrence, Chemotherapy, Neoadjuvant treatment

## Abstract

**Background:**

Early recurrence after esophagectomy is often used as a surrogate for aggressive tumor biology and treatment failure. However, there is no standardized definition of early recurrence, and predictors for early recurrence are unknown. Therefore, we aimed to define an evidence-based cutoff to discriminate early and late recurrence and assess the influence of neoadjuvant treatment modalities for patients with esophageal or gastroesophageal-junction adenocarcinoma (EAC).

**Patients and Methods:**

This dual-center retrospective cohort study included patients who underwent esophagectomy for stage II–III EAC after neoadjuvant treatment with chemotherapy using 5-fluorouracil, leucovorin, oxaliplatin, and docetaxel (FLOT) or radiochemotherapy according to the Chemoradiotherapy for Esophageal Cancer followed by Surgery Study (CROSS) protocol from 2012 to 2022. The optimal cutoff for early versus late recurrence was calculated by using the most significant difference in survival after recurrence (SAR). Multivariable logistic regression was used to identify variables associated with early recurrence.

**Results:**

Of 334 included patients, 160 (47.9%) were diagnosed with recurrence. Most patients had systemic (60.5%) or multiple sites of recurrence (21.1%), whereas local-only recurrence (9.2%) and carcinomatosis (9.2%) were rare. The optimal interval between surgery and recurrence for distinguishing early and late recurrence was 18 months (median SAR: 9.1 versus 17.8 months, *p* = 0.039) with only 24% of recurrences diagnosed after the calculated cutoff. Advanced pathologic tumor infiltration (ypT3–4, *p* = 0.006), nodal positivity (*p* = 0.013), poor treatment response (>10% residual tumor, *p* = 0.015), and no adjuvant treatment (*p* = 0.048) predicted early recurrence.

**Conclusion:**

Early recurrence can be defined as recurrent disease within 18 months. Hallmarks for early recurrence are poor response to neoadjuvant therapy with persisting advanced disease. In those patients, adjuvant therapy and closer follow-up should be considered.

**Supplementary Information:**

The online version contains supplementary material available at 10.1245/s10434-024-16403-5.

Esophageal adenocarcinoma (EAC) accounts for 80% of esophageal malignancies in the Western world.^1^ With only 21.7% 5-year survival across all stages, it portends the third worst relative survival rate, after pancreatic and liver cancer.^1^ This dismal prognosis is mainly driven by early dissemination of disease, leading to a significant number of patients suffering from metastatic disease at time of presentation.^2^ In patients who are diagnosed with resectable disease, the introduction of neoadjuvant therapy has led to a modest improvement in overall survival (OS) and time to progression (TTP).^3^ Currently, multimodal regimens such as FLOT and CROSS are routinely used in a neoadjuvant approach for local and systemic control of disease.^4,5^ However, a significant number of patients develop treatment resistance, as evidenced by a major histopathologic response rate of only 37% and recurrence in approximately one out of two resected EAC patients after neoadjuvant treatment.^6–8^

Early recurrence is often used as a surrogate marker for aggressive tumor biology and treatment failure. As shown in pancreatic cancer, early recurrence is associated with impaired survival after recurrence.^9^ However, a clear and statistically sound definition for early recurrence in esophageal adenocarcinoma is lacking. In the literature, arbitrary cutoffs of 6 or 12 months are used.^10,11^ As early recurrence has important implications for patient prognosis, accurate prediction of early recurrence could aid treatment decisions in patients with poor performance status and high risk of early recurrence. In such patients, the risk–benefit balance of treatments with significant morbidity and mortality could be avoided.^12^ Furthermore, a high propensity for early recurrence could indicate the need for continued or adjuvant systemic treatment in patients after esophagectomy or could guide follow-up post esophagectomy cancer.^13^

The aim of this study is to define early recurrence by the difference in survival after recurrence and assess the influence of neoadjuvant treatment modalities on early recurrence. Furthermore, we aim to define clinical and pathological predictors of early recurrence and for implications of postsurgical treatment and follow-up.

## PATIENTS AND METHODS

### *Study Design*

This study presents a multicenter cohort analysis of surgically treated patients with esophageal or gastroesophageal adenocarcinoma after neoadjuvant treatment. Data were extracted from two prospectively managed databases. The study was performed in compliance with the Strengthening the Reporting of OBservational studies in Epidemiology (STROBE) guidelines.^14^ The study was approved by the Ethics Committee of Heidelberg University (S-649-2012) and complied with the 1964 Helsinki Declaration and its later amendments. All included patients filed informed consent for data use upon treatment.

### *Participants*

Patients with EAC who underwent surgical resection with curative intent between 2012 and 2021 at Heidelberg University Hospital, Germany and the Clarunis University Digestive Health Care Center in Basel, Switzerland were included. Patients with clinically staged UICC stage I disease were excluded.^15^ Therefore, only patients with an indication for neoadjuvant therapy according to European Society for Medical Oncology (ESMO) and National Comprehensive Cancer Network (NCCN) guidelines were included.^16,17^ Further exclusion criteria encompassed pathologically proven synchronous metastatic disease, macroscopic positive resection margin (R2), neoadjuvant treatment with regimens other than FLOT (5-fluorouracil, leucovorin, oxaliplatin, and docetaxel) or platinum-based radiochemotherapy (CROSS), 90-day mortality without recurrence, and inadequate follow-up data. Inadequate follow-up data was defined as loss to follow-up without event (recurrence or death) within 12 months and incomplete medical records for the primary outcomes, e.g., owing to follow-up at another institution.

### *Treatment*

Detailed treatment algorithms are described elsewhere.^18^ In brief, treatment decisions including mode of neoadjuvant therapy and type of surgery were made in an interdisciplinary tumor board. The decision on the treatment plan was made independently of this study. Neoadjuvant (radio)chemotherapy was administered with either four cycles of FLOT or platinum-based radiochemotherapy according to the CROSS protocol. Staging was performed by endoscopy and cross-sectional imaging [computed tomography (CT), magnetic resonance imaging (MRI), and/or positron emission tomography (PET)-CT] before and after neoadjuvant treatment.

Surgery was performed by abdominothoracic esophagectomy with Ivor-Lewis reconstruction (ILE) for cancers proximal to the gastroesophageal junction (GEJ) or transhiatal esophagectomy with total gastrectomy and Roux-Y reconstruction (THG) in GEJ Siewert type 3 cancers based on upper endoscopy results.^19^ The decision regarding ILE or THG in GEJ 2 cancers was based on randomization under study conditions or surgeon preference.^20^ Signet ring cell positive cancers with known extension to the stomach were preferably treated with THG. A gastric conduit was routinely used for reconstruction in ILE, with a colon conduit as an alternative when reconstruction with the former method was not possible.

### *Follow-Up and Data Collection*

Patients were followed up with regular clinical visits, serologic, radiographic, and endoscopic diagnostics every 3 months within the first 2 years after surgery and every 6 months thereafter until 60 months postoperatively. Site of recurrence was extracted from results of CT or MRI imaging, endoscopy with or without ultrasound, and biopsies as well as cytologic analyses of peritoneal fluid, where applicable. Further follow-up data were obtained through electrical records of clinical visits, telephone interviews with patients or their primary care provider, and death certificates.

### *Outcomes*

The American Joint Committee on Cancer (AJCC)/Union for International Cancer Control (UICC) 8th edition was used for tumor–node–metastasis (TNM) staging.^15^ Treatment response was assessed according to the Becker or Mandard classification.^7,21^ Becker 1a and Mandard 1 (complete regression) as well as Becker 1b (< 10% vital tumor cells) and Mandard 2 (rare residual cancer cells scattered through the fibrosis) were defined as major histopathologic treatment response. Overall survival (OS) was defined as time from diagnosis to death. When time of diagnosis was not available, the starting date of neoadjuvant treatment was used. Time to progression (TTP) was defined as time from surgery to cancer recurrence, and survival after recurrence (SAR) was defined as time of diagnosis of recurrence to time of death. Sites of recurrence were defined as local, carcinomatosis, or systemic recurrence. If multiple of the aforementioned sites were involved, then the recurrence site was stratified as multiple. Systemic recurrence encompassed all distant recurrence locations including liver, lung, nonregional lymph nodes, bone, brain, adrenal gland, muscles, skin, or multiple of these sites. Carcinosis recurrence encompassed peritoneal and pleural carcinosis as well as Krukenberg tumors. Local recurrence was defined as intra- or extraluminal locoregional recurrence.

### *Statistical Analysis*

Statistical analysis was performed using R statistical software (version 4.2.0). The Survival and Survminer packages were used for Kaplan–Meier and logistic regression analyses. Ggplot2 and Forester were used for data visualization. A two-sided *p*-value of < 0.05 was considered statistically significant.

Differences in the distribution of categorical data were compared using the chi-squared test, and continuous data were compared using the Wilcox rank-sum test. The Kruskal–Wallis test was used for comparisons of multiple groups with nonparametric data. Missing data are mentioned in the tables but removed for group comparisons. The optimal cutoff to differentiate early from late recurrence was calculated by using the most significant difference in SAR as described by Hothorn et al.^22^ This method uses a repetitive approach where, at each instance, a specific time to recurrence is used to split the patient population into those who have a value below and above the tested cutoff value. For each cutoff, the association with the outcome (in this case, SAR) is examined. The optimal cutoff point corresponds to the value of the variable that results in the best discrimination of SAR. Another method used in medical literature is manually searching for the lowest *p*-value between the two respective groups. Uni- and multivariable logistic regression analyses with backward elimination were used to identify variables associated with early recurrence. Hazard ratios (HRs) and 95% confidence intervals (95% CIs) were calculated for each pretreatment-assessed variable and post-surgery available variable separately. Survival comparisons are visualized using Kaplan–Meier curves. Subgroup analyses were performed for different treatment regimens.

## RESULTS

### *Patient Cohort*

A total of 968 patients received surgical treatment for esophageal and gastroesophageal cancer of any type at the Heidelberg University Hospital or the Clarunis University Digestive Health Care Center, Basel between 2012 and 2021. Of those, 573 were treated for adenocarcinoma with UICC stage II or III. After serial exclusion due to not having received neoadjuvant treatment (*N* = 78), treatment regimen other than FLOT or CROSS (*N =* 47), 90-day mortality (*N* = 20), or insufficient data or loss to follow-up (*N* = 96), a total of 334 patients were included in the final analysis. Median follow-up was 38.4 months for surviving patients. Eighty-four percent were treated with FLOT, and 16% with CROSS. Other clinicopathological characteristics are presented in Table 1.

**Table 1:** Baseline characteristics for all patients and stratified by recurrence status

	All patients, *N* = 334^1^	No recurrence, *N* = 174^1^	Recurrence, *N* = 160^1^	*p*-Value^*2*^
Age (years)	62.50 (10.54)	61.76 (10.45)	62.08 (10.68)	0.856
Sex				0.847
Male	279 (84%)	146 (84%)	133 (83%)	
Female	55 (16%)	28 (16%)	27 (17%)	
Location				0.606
GEJ 1 and above	164 (49%)	87 (50%)	77 (48%)	
GEJ 2	132 (40%)	65 (37%)	67 (42%)	
GEJ 3	38 (11%)	22 (13%)	16 (10%)	
Grade				0.512
Well–moderate	144 (48%)	77 (50%)	67 (46%)	
Poor	155 (52%)	77 (50%)	78 (54%)	
Signet ring cells				0.053
Negative	252 (86%)	136 (90%)	116 (82%)	
Positive	40 (14%)	15 (9.9%)	25 (18%)	
cT-Stage				0.229
cT1/2	35 (11%)	22 (13%)	13 (8.7%)	
cT3/4	284 (89%)	148 (87%)	136 (91%)	
cN-Stage				0.178
cN0	35 (10%)	22 (13%)	13 (8.1%)	
cN+	299 (90%)	152 (87%)	147 (92%)	
Type of neoadjuvant treatment				0.379
FLOT	282 (84%)	144 (83%)	138 (86%)	
Radiochemotherapy	52 (16%)	30 (17%)	22 (14%)	
Type of surgery				0.774
THG	88 (26%)	47 (27%)	41 (26%)	
ILE	246 (74%)	127 (73%)	119 (74%)	
Complications	118 (35%)	58 (33%)	60 (38%)	0.426
pT-Stage				**< 0.001**
pT0–2	123 (37%)	86 (49%)	37 (23%)	
pT3–4	211 (63%)	88 (51%)	123 (77%)	
pN-Stage				**< 0.001**
pN0	149 (45%)	101 (58%)	48 (30%)	
pN+	185 (55%)	73 (42%)	112 (70%)	
Resection Margin				0.528
R0	308 (92%)	162 (93%)	146 (91%)	
R1	26 (7.8%)	12 (6.9%)	14 (8.8%)	
Regression				**<0.001**
Major response	111 (35%)	80 (48%)	31 (20%)	
Moderate–no response	208 (65%)	86 (52%)	122 (80%)	
Adjuvant treatment	207 (66%)	111 (69%)	96 (64%)	0.316

Significant values presented in bold

^1^Data presented as *n* (%) and mean (SD)

^2^Pearson’s chi-squared test; Wilcoxon rank sum test; Fisher’s exact test for no recurrence versus recurrence

*GEJ* gastroesophageal junction according to the Siewert classification, *THG* total gastrectomy with transhiatal distal esophagectomy, *ILE* Ivor-Lewis esophagectomy

Regression: major response defined as tumor regression grade 1a and 1b (Becker classification)

### *Recurrence*

**Fig. 1:**
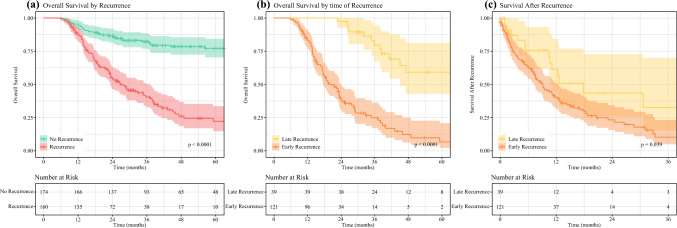
Kaplan–Meier analysis for overall survival and survival after recurrence. Kaplan–Meier plots for (**a**) overall survival in patients with versus patients without recurrence, (**b**) overall survival in patients with early versus late recurrence, and (**c**) survival after recurrence for patients with early versus late recurrence. *p*-Value derived by log-rank test; 95% confidence interval indicated by hatched area

The median TTP of the whole cohort was 44.1 months (95% CI: 38.8 months to not reached). Of 334 included patients, 160 (48%) were diagnosed with recurrence after a median of 8.8 months (95% CI 7.0–9.9 months) after surgery. Median SAR was 10.5 months (95%CI 8.0–12.7 months). The remaining 52% had no recurrence after median follow-up of 37.4 months. Demographic and clinicopathological characteristics for patients with and without recurrence are summarized in Table 1. Notably, no preoperatively available variable was associated with recurrence. However, significant associations with recurrence were found for postoperative pathological pT-Stage (*p* < 0.001), pN-Stage (*p* < 0.001), lymph node ratio (*p* < 0.001), and response to neoadjuvant treatment (*p* < 0.001). Even after complete pathological response of the primary tumor, recurrence was observed in 18% of patients (Supplementary Fig. 1). Recurrence was diagnosed as local recurrence in 9.2%, carcinomatosis in 9.2%, and systemic in 60.5%. The other 21.1% had multiple recurrence sites involved. Patients with recurrence had significantly shorter OS as compared with patients without recurrence (median OS: 27.7 months versus not reached, *p* < 0.001; Fig. 1a). Consequently, the estimated 5-year survival for patients with recurrence was lower at 22%, versus 77% for patients without recurrence (*p* < 0.001). Early recurrence was diagnosed in 35% after conventional CT and 41% who received additional PET-CT for presurgical staging of disease (*p* = 0.416).

### *Definitions for Early and Late Recurrence*

**Fig. 2:**
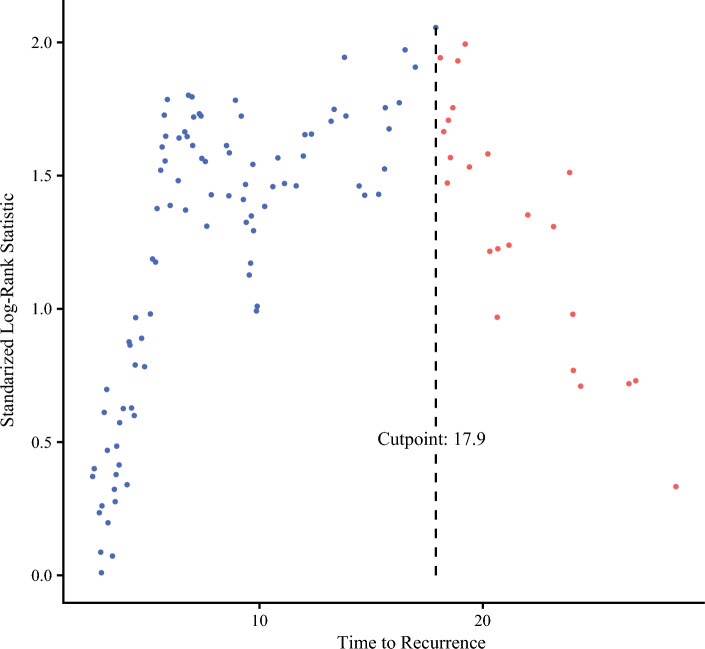
Defining early versus late recurrence by maximally selected log-rank statistic. Dots represent standardized log-rank estimates of survival after recurrence with a maximum between 17.9 and 18.0 months after surgery, as indicated by the dashed line. Similar to the lowest *p*-value method, this indicates the most significant cutoff for separating early and late recurrence

In this study, the optimal length to distinguish early from late recurrence was 17.9 months (Fig. 2). Patients with early recurrence (*N* = 121) had shorter median OS compared with patients with late recurrence (*N* = 39) (19.9 versus 71.2 months, *p* < 0.001; Fig. 1b) and, by definition, a shorter median SAR with 9.1 months in the early and 17.8 months in the late recurrence cohort (*p* = 0.039; Fig. 1c).

Compared with late recurrence, early recurrence was associated with preoperative clinically node positive disease (95% versus 82%, *p* = 0.017) and a higher lymph node ratio (mean 0.21 versus 0.09, *p* = 0.010; Table 2). There were no significant differences for patterns of recurrence in early versus late recurrence, with 8.8% versus 10% local recurrences, 9.7% versus 7.7% peritoneal recurrences, 58% versus 67% systemic recurrences, and 23% versus 15% with multiple-site recurrence (*p* = 0.726). There was also no significant difference in the rate of treatment received for recurrence in the early versus late cohorts (93.5% versus 86.3%, *p* = 0.275).

**Table 2:** Comparison of early and late recurrence (≥ 18 months)

	Early recurrence, *N* = 121^1^	Late recurrence *N* = 39^1^	*p*-Value^2^
Age (years)	62.38 (10.76)	61.15 (10.49)	0.527
Sex			0.485
Male	102 (84%)	31 (79%)	
Female	19 (16%)	8 (21%)	
Tumor location			0.229
GEJ 1 and above	57 (47%)	20 (51%)	
GEJ 2	49 (40%)	18 (46%)	
GEJ 3	15 (12%)	1 (2.6%)	
Grade			0.971
Well–moderate	50 (46%)	17 (46%)	
Poor	58 (54%)	20 (54%)	
Signet ring cells			0.309
Negative	90 (84%)	26 (76%)	
Positive	17 (16%)	8 (24%)	
cT-Stage			0.318
cT1/2	8 (7.2%)	5 (13%)	
cT3/4	103 (93%)	33 (87%)	
cN-Stage			**0.017**
cN0	6 (5.0%)	7 (18%)	
cN+	115 (95%)	32 (82%)	
Type of neoadjuvant treatment			0.846
FLOT	104 (86%)	34 (87%)	
Radiochemotherapy	17 (14%)	5 (13%)	
Type of surgery			0.400
THG	33 (27%)	8 (21%)	
ILE	88 (73%)	31 (79%)	
Complications	45 (37%)	15 (38%)	0.887
pT-Stage			0.082
pT0–2	24 (20%)	13 (33%)	
pT3–4	97 (80%)	26 (67%)	
pN-Stage			0.185
pN0	33 (27%)	15 (38%)	
pN+	88 (73%)	24 (62%)	
Lymph node ratio	0.21 (0.23)	0.09 (0.11)	**0.010**
Resection margin			0.191
R0	108 (89%)	38 (97%)	
R1	13 (11%)	1 (2.6%)	
Regression			0.100
Major response	20 (17%)	11 (30%)	
Moderate–no response	96 (83%)	26 (70%)	
Adjuvant treatment	70 (61%)	26 (70%)	0.330

Significant values presented in bold

^1^Data presented as *n* (%) and mean (SD)

^2^Pearson’s Chi-squared test; Wilcoxon rank-sum test; Fisher’s exact test for early versus late recurrence

*GEJ* gastroesophageal junction according to the Siewert classification, *THG* total gastrectomy with transhiatal distal esophagectomy, *ILE* Ivor-Lewis esophagectomy

Regression: major response defined as tumor regression grade 1a and 1b (Becker classification)

### *Predictors of Early Recurrence*

The early recurrence cohort was compared with the patients who had either late or no recurrence. The results of univariate logistic regression analysis are presented in Table 3 and were calculated for presurgically and postoperative available variables separately in the multivariable analysis. Notably, in pretreatment available variables, only clinically positive nodal status at time of diagnosis was a predictor of early recurrence (OR 3.02, 95% CI:1.22–7.50, *p* = 0.017). The excellent positive predictive value of 95% is, however, opposed by the poor negative predictive value of 18%. Univariate analysis of postoperatively available variables yielded significant associations of early recurrence to advanced pathological tumor extension (pT3–4: OR 3.51, 95% CI: 2.08–5.91, *p* < 0.001), nodal positive disease (reference pN0, OR 3.19, 95% CI: 1.97–5.17, *p* < 0.001), insufficient response to neoadjuvant treatment (reference Mandard TRG 1–2, OR 3.90, 95% CI: 2.23–6.80, *p* < 0.001). On multivariable analysis, advanced T-stage (pT3–4, adjusted OR 2.52, 95% CI: 1.32–4.91, *p* = 0.006), nodal positive disease (pN1–pN3, adjusted OR 2.06, 95% CI: 1.17–3.66, *p* = 0.013), minor histopathological response (> 10% residual tumor, adjusted OR 2.25, 95% CI: 1.18–4.40, *p* = 0.015), and no adjuvant therapy (adjusted OR 1.74, 95% CI: 1.01–3.04, *p* = 0.048) predicted early recurrence.

**Table 3:** Univariable logistic regression analysis for predictors of early recurrence

	Variable	Odds ratio (95% CI)	*p*-Value
Age	< 65 years	Reference	
	> 65 years	1.09 (0.69–1.70)	0.720
ASA	1/2	Reference	
	3/4	1.02 (0.65–1.62)	0.922
Sex	Male	Reference	
	Female	0.92 (0.50–1.68)	0.776
Neoadjuvant treatment	FLOT	Reference	
	CROSS	0.83 (0.44–1.56)	0.564
Location	GEJ 1 and above	Reference	
	GEJ 2	1.11 (0.69–1.79)	0.673
Grade	Well–moderate	Reference	
	Poor	1.12 (0.70–1.80)	0.628
Signet ring cells	Negative	Reference	
	Positive	1.33 (0.68–2.62)	0.409
cT-Stage	1/2	Reference	
	3/4	1.92 (0.84–4.38)	0.121
cN-Stage	Negative	Reference	
	Positive	3.02 (1.22–7.50)	**0.017**
Type of surgery	THG	Reference	
	ILE	0.93 (0.56–1.54)	0.772
pT-Stage	1/2	Reference	
	3/4	3.51 (2.08–5.91)	**< 0.001**
pN-Stage	N0	Reference	
	N+	3.19 (1.97–5.17)	**< 0.001**
Resection Margin	R0	Reference	
	R1	1.85 (0.83–4.14)	0.133
Regression	Major	Reference	
	Poor	3.90 (2.24–6.80)	**< 0.001**
Adjuvant treatment	Yes	Reference	
	No	1.41 (0.87–2.29)	0.162

Patients with early recurrence compared with late or no recurrence

Significant values presented in bold

### *Subanalyses for Type of Chemotherapy and Type of Surgery*

Patients treated with FLOT were significantly younger (*p* = 0.007), had more distal tumor location (*p* < 0.001), and were therefore more often treated with gastrectomy with transhiatal distal esophagectomy (*p* < 0.001, Supplementary Table 1). Furthermore, major complications (Clavien–Dindo ≥ 3) were observed more often after treatment with FLOT compared with CROSS (38% versus 23%, *p* = 0.044). Adjuvant treatment was more often given to patients after FLOT (76% versus 19%, *p* < 0.001), with 175 patients (92%) receiving additional cycles of FLOT and 15 (8%) radiochemotherapy. Despite no significant differences in clinically staged T and N stages, histopathological analyses yielded more advanced T stage (*p* = 0.032) and more nodal positive disease (*p* = 0.018) in the FLOT cohort. The R1 resection rate was similar in the two cohorts (R1: 7.8% versus 7.7%, *p* = 0.999). However, the treatment regimen was not associated with the occurrence of early recurrence (37% versus 33%, *p* = 0.564) or recurrence overall (49% versus 42%, *p* = 0.379). Local recurrence was observed in 8.7% of the FLOT group versus 2.0% of the CROSS group (*p* = 0.095), while peritoneal recurrence was observed in 11.1% and 19.6% (*p* = 0.091), respectively. There was no difference in the occurrence of systemic recurrence between the two regimens (37.6% versus 37.3%, *p* = 0.959).

Comparisons for gastrectomy with transhiatal distal esophagectomy versus transthoracic esophagectomy with Ivor-Lewis reconstruction yielded significant differences in tumor location (*p* < 0.001), type of neoadjuvant treatment (*p* < 0.001), and pathological nodal positive disease (*p* = 0.039, Supplementary Table 2). The resection margin was positive in 13% after THG compared with 6.1% after ILE (*p* = 0.054). However, again, there was no difference in the occurrence of early recurrence (38% versus 36%, *p* = 0.772) or recurrence overall (47% versus 48%, *p* = 0.774).

## DISCUSSION

In this analysis, an evidence-based cutoff for separating early from late recurrence was found at 1.5 years after surgical resection. Patients with early recurrence had significantly shorter survival after recurrence and consequently much shorter overall survival compared with patients after late recurrence. Independent risk factors for early recurrence encompassed pathological advanced T stage, nodal positive disease, minor histopathological response, and no adjuvant therapy. However, except for nodal positivity at time of diagnosis, no variables that are known prior to surgical treatment could predict early recurrence. Except from providing adjuvant therapy, treatment modalities such as type of neoadjuvant treatment or surgical approach have no influence on early recurrence and recurrence overall.

In the literature, varying cutoffs, such as 6 or 12 months, are used for early recurrence in esophageal cancer.^10,11^ To our knowledge, this is the first evidence-based statistical cutoff to be defined for this cancer entity. This optimal time from surgery to recurrence to separate early and late recurrence was defined by determining the best discrimination in SAR using log-rank testing, similar to the minimum *p* value approach. While the value of the cutoff derived from the unique dataset used may be influenced by patient numbers and treatment modalities and center-specific follow-up strategies, this multicenter study is representative for Western high-volume centers. Interestingly, the more aggressive the gastrointestinal tumor entity, the shorter the time to recurrence.^1^ With 18 months, the cutoff for esophageal adenocarcinoma is higher as compared with pancreatic cancer with 12 months as defined by Groot et al. or liver cancer with 8 months as defined by Xing et al.^9,23^ For other gastrointestinal tumor entities, similar or higher cutoffs are described in literature: 16 months for colon cancer, 21 months for esophageal squamous cell carcinoma, and 24 months for rectal cancer after neoadjuvant treatment.^24–26^

Early recurrence is often perceived as failure of surgery, and therefore the value of surgical resection is questioned by both the patient and the physician. Consequently, predictors of early recurrence could aid in treatment decisions, especially in those patients with questionable fitness to endure the surgical procedure and expected prolonged postoperative recovery.^8,27^ However, the only preoperative available predictor of early recurrence is pretreatment clinical nodal positivity, with a positive predictive value of 95%, meaning that, if present, early recurrence is likely. However, for clinical decision-making, some limitations of this measure must be considered. First, the negative predictive value is poor at only 18%. Second, as shown in other analyses, clinical nodal staging with computer tomography is imprecise.^8,28,29^ Enlargement of lymph nodes beyond the cutoff of 1 cm is deemed as suspicious for lymph node involvement of cancer. Since enlargement of lymph nodes can also be caused by peritumoral inflammation, while metastatic lymph nodes can measure below the cutoff value of 10 mm, poor performance of CT scans in terms of diagnostic accuracy (38–77%) and sensitivity (14–24%) is seen in clinical practice.^30^ Therefore, false-negative results are often present when comparing clinical with pathological nodal positivity. In the future, more adequate lymph node examinations, for example, through biopsies or more frequent use of PET-CT scans and liquid biopsies as a marker for systemic disease, may fill the gap and could influence the necessity of cytotoxic treatment or guide decision-making in borderline fit patients with high probability of early systemic recurrence.^31^ Importantly, the surgical approach, mode of neoadjuvant treatment, and use of PET-CT for clinical staging had no influence on the occurrence of early recurrence or recurrence overall. Also, there was no difference in recurrence patterns for early versus late recurrence or within the treatment regimens studies.

In the absence of valid serum tumor markers for surveillance after esophagectomy, many institutions alternate CT imaging with upper endoscopy every 3 months within the first 2 years and every 6 months thereafter until the fifth postoperative year for every patient.^32,33^ While unadjusted for tumor biology, some patients may not profit from such close follow-up that may cause a psychological burden for the patient and healthcare costs for society.^34^ On the other hand, those with high propensity for early recurrence could benefit from close follow-up and early change of treatment strategies. Early detection of recurrence could especially result in more curatively intended treatments before further progression of the disease.^35^ In this analysis, postoperatively advanced T stage, nodal positive disease, minor histopathological response, and no adjuvant treatment were predictors for early recurrence. In addition, positive resection margin and poor tumor differentiation can be found as predictors for recurrence in literature.^10,36,37^ In such patients, an intensified adjuvant therapy or even regimen switch compared with the preoperative neoadjuvant therapy may be considered together with close follow-up.

As this was a retrospective analysis, it is associated with potential bias corresponding to the study design. While the aforementioned poor prognostic factors are indicative of unfavorable outcomes, it is imperative not to adopt an exclusively pessimistic outlook if one or multiple factors are present. On the other hand, their absence does not preclude unfavorable outcomes, as shown by some early recurrences despite complete pathologic response of the primary tumor in this study. Furthermore, selection of neoadjuvant therapy was based on institutional preference for FLOT in one center and radiochemotherapy in the other center. Clear trends are shown favoring less locoregional but more peritoneal recurrences in CROSS. While not the primary goal of this study, the limited number of patients treated with CROSS may have produced a false-negative result. However, large studies comparing CROSS versus FLOT also found equal recurrence rates and no clear differences for better local control via radiotherapy nor better systemic control via aggressive systemic treatment with FLOT.^38–40^ Contrarily, although no recurrence rates and sites have been published yet, the recently presented ESOPEC trial suggests improved survival for FLOT compared with CROSS.^41^ Furthermore, the exclusion of patients without sufficient follow-up or who received neoadjuvant chemotherapy regimens other than the currently used regimens of CROSS and FLOT may limit the real-world applicability to a referral center with ongoing trials, including ours. However, by doing so, we ensure a uniform patient cohort with reliable timepoint of recurrence and treatment generalizable to current clinical practice. Promising results have been shown for PD-1-directed immunotherapy in the neoadjuvant setting, with increased rates of major pathological treatment response and lower recurrence rates when given adjuvantly.^42,43^ In the future, treatment with PDL-1 inhibitors may become standard of care and could alter the prognostic relevance of some established pathologic features.

## CONCLUSIONS

This is the first study to provide an evidence-based cutoff for early recurrence in patients after resection of esophageal adenocarcinoma. Early recurrence can be defined as recurrent disease within 18 months based on SAR. Hallmarks for early recurrence are poor response to neoadjuvant therapy with remaining advanced disease (ypT3/4, ypN+) indicative of unfavorable tumor biology. However, these factors cannot be reliably predicted before resection. Importantly, the type of neoadjuvant treatment has no influence on timing of recurrence and recurrence patterns. In patients with remaining advanced disease after resection, additional adjuvant therapy and shorter follow-up times should be considered.

## Supplementary Information

Below is the link to the electronic supplementary material.

Supplementary file1 (DOCX 1071 KB)
