# Supplementary material for: Definition and Predictors of Early Recurrence in Neoadjuvantly Treated Esophageal and Gastroesophageal Adenocarcinoma: a Dual-Center Retrospective Cohort Study
Source: Ann Surg Oncol. 2024 Nov 5;32(3):1617–27. doi: 10.1245/s10434-024-16403-5 (PMC11811458; doi:10.1245/s10434-024-16403-5)
Supplement: Supplementary file 1 — Supplementary file1 (DOCX 1071 KB) [file 10434_2024_16403_MOESM1_ESM.docx]

| Supplementary Table 1: FLOT vs. CROSS | FLOT,  N = 282*^1^* | Radio-chemotherapy, N = 52*^1^* | p-value*^2^* |
| --- | --- | --- | --- |
| ASA |  |  | 0.052 |
| 1/2 | 120 (43%) | 15 (29%) |  |
| 3/4 | 157 (57%) | 37 (71%) |  |
| Unknown | 5 | 0 |  |
| Age (>65) | 111 (39%) | 31 (60%) | 0.007 |
| Sex (female) | 47 (17%) | 8 (15%) | 0.819 |
| Location | |  | <0.001 |
| GEJ 1 and above | 123 (44%) | 41 (79%) |  |
| GEJ 2 | 122 (43%) | 10 (19%) |  |
| GEJ 3 | 37 (13%) | 1 (1.9%) |  |
| Grade |  |  | 0.248 |
| well-moderate | 125 (50%) | 19 (40%) |  |
| poor | 127 (50%) | 28 (60%) |  |
| Unknown | 30 | 5 |  |
| Signet ring cells | |  | 0.722 |
| negative | 237 (86%) | 15 (83%) |  |
| positive | 37 (14%) | 3 (17%) |  |
| Unknown | 8 | 34 |  |
| cT_Stage | |  | 0.492 |
| cT1/2 | 28 (10%) | 7 (14%) |  |
| cT3/4 | 240 (90%) | 44 (86%) |  |
| Unknown | 14 | 1 |  |
| cN |  |  | 0.08 |
| cN0 | 26 (9.2%) | 9 (17%) |  |
| cNpositive | 256 (91%) | 43 (83%) |  |
| Type of Surgery | |  | <0.001 |
| THG | 87 (31%) | 1 (1.9%) |  |
| ILE | 195 (69%) | 51 (98%) |  |
| Complications | 106 (38%) | 12 (23%) | 0.044 |
| pT-Stage | |  | 0.032 |
| pT0-2 | 97 (34%) | 26 (50%) |  |
| pT3-4 | 185 (66%) | 26 (50%) |  |
| pN-Stage | |  | 0.018 |
| pN0 | 118 (42%) | 31 (60%) |  |
| pN1 | 164 (58%) | 21 (40%) |  |
| Lymph Node Ratio | 0.14 (0.20) | 0.08 (0.16) | 0.013 |
| Resection Margin | |  | >0.999 |
| R0 | 260 (92%) | 48 (92%) |  |
| R1 | 22 (7.8%) | 4 (7.7%) |  |
| Regression | |  | 0.214 |
| good response | 89 (33%) | 22 (42%) |  |
| moderate-no response | 178 (67%) | 30 (58%) |  |
| Unknown | 15 | 0 |  |
| Adjuvant Tx | 197 (76%) | 10 (19%) | <0.001 |
| Unknown | 22 | 0 |  |

| Supplementary Table 2: Comparisons of Surgical Approches | THG, N = 88*^1^* | ILE, N = 246*^1^* | p-value*^2^* |
| --- | --- | --- | --- |
| ASA |  |  | 0.939 |
| 1/2 | 36 (41%) | 99 (41%) |  |
| 3/4 | 51 (59%) | 143 (59%) | |
| Unknown | 1 | 4 |  |
| Age |  |  | 0.249 |
| ≤65 | 46 (52%) | 146 (59%) | |
| >65 | 42 (48%) | 100 (41%) | |
| Sex |  |  | 0.401 |
| male | 71 (81%) | 208 (85%) | |
| female | 17 (19%) | 38 (15%) |  |
| Location | |  | <0.001 |
| GEJ 1 and above | 1 (1.1%) | 163 (66%) | |
| GEJ 2 | 55 (63%) | 77 (31%) |  |
| GEJ 3 | 32 (36%) | 6 (2.4%) |  |
| Grade |  |  | 0.329 |
| well-moderate | 32 (43%) | 112 (50%) | |
| poor | 42 (57%) | 113 (50%) | |
| Unknown | 14 | 21 |  |
| Signet ring cells | |  | 0.103 |
| negative | 69 (81%) | 183 (88%) | |
| positive | 16 (19%) | 24 (12%) |  |
| Unknown | 3 | 39 |  |
| cT_Stage | |  | 0.93 |
| cT1/2 | 9 (11%) | 26 (11%) |  |
| cT3/4 | 75 (89%) | 209 (89%) | |
| Unknown | 4 | 11 |  |
| cN |  |  | 0.62 |
| cN0 | 8 (9.1%) | 27 (11%) |  |
| cNpositive | 80 (91%) | 219 (89%) | |
| Type of Neoadjuvant Tx | | | <0.001 |
| FLOT | 87 (99%) | 195 (79%) | |
| Radio-chemotherapy | 1 (1.1%) | 51 (21%) |  |
| LKentf | 29.28 (12.53) | 27.75 (10.06) | 0.495 |
| Complications | 25 (28%) | 93 (38%) | 0.114 |
| pT-Stage | |  | 0.056 |
| pT0-2 | 25 (28%) | 98 (40%) |  |
| pT3-4 | 63 (72%) | 148 (60%) | |
| pN-Stage | |  | 0.039 |
| pN0 | 31 (35%) | 118 (48%) | |
| pN1 | 57 (65%) | 128 (52%) | |
| Lymph Node Ratio | 0.19 (0.25) | 0.11 (0.17) | 0.006 |
| Unknown | 1 | 0 |  |
| Resection Margin | |  | 0.054 |
| R0 | 77 (88%) | 231 (94%) | |
| R1 | 11 (13%) | 15 (6.1%) | |
| Regression | |  | 0.299 |
| good response | 26 (30%) | 85 (36%) |  |
| moderate-no response | 60 (70%) | 148 (64%) | |
| Unknown | 2 | 13 |  |
| Adjuvant Tx | 58 (73%) | 149 (64%) | 0.124 |
| Unknown | 9 | 13 |  |

**Supplementary Figure 1:**


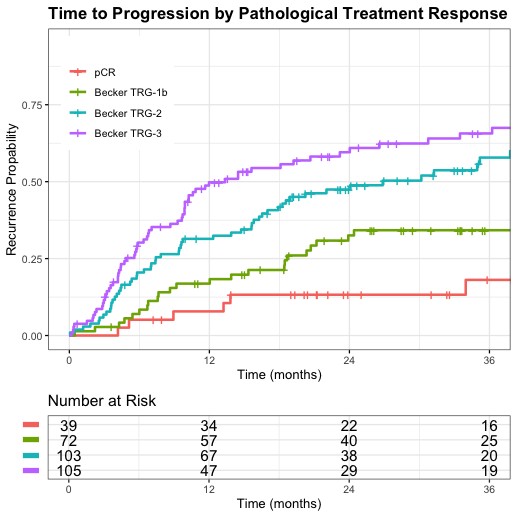


| Becker TRG | N | Recurrence | Estimated 3-year [95%CI] | Early Recurrence | Late Recurrence |
| --- | --- | --- | --- | --- | --- |
| 1a (pCR) | 39 | 7 (18%) | 18% [3-31%] | 5 (13%) | 2 (5%) |
| 1b | 72 | 24 (33%) | 34% [22-45%] | 15 (21%) | 9 (13%) |
| 2 | 103 | 57 (55%) | 58% [45-68%] | 42 (41%) | 15 (15%) |
| 3 | 105 | 65 (62%) | 66% [54-74%] | 54 (51%) | 11 (10%) |
